# Sequential stopping for high-throughput experiments

**DOI:** 10.1093/biostatistics/kxs026

**Published:** 2012-08-20

**Authors:** David Rossell, Peter Müller

**Affiliations:** Biostatistics and Bioinformatics Unit, Institute for Research in Biomedicine of Barcelona, Barcelona 08028, Spain; Department of Mathematics, UT Austin, Austin 78712, TX, USA

**Keywords:** Decision theory, Forward simulation, High-throughput experiments, multiple testing, Optimal design, Sample size, Sequential design

## Abstract

In high-throughput experiments, the sample size is typically chosen informally. Most formal sample-size calculations depend critically on prior knowledge. We propose a sequential strategy that, by updating knowledge when new data are available, depends less critically on prior assumptions. Experiments are stopped or continued based on the potential benefits in obtaining additional data. The underlying decision-theoretic framework guarantees the design to proceed in a coherent fashion. We propose intuitively appealing, easy-to-implement utility functions. As in most sequential design problems, an exact solution is prohibitive. We propose a simulation-based approximation that uses decision boundaries. We apply the method to RNA-seq, microarray, and reverse-phase protein array studies and show its potential advantages. The approach has been added to the Bioconductor package gaga.

## 1. Introduction

In high-throughput studies (HTSs), the sample size is usually chosen informally. The resulting experiment may either be not informative enough or unnecessarily extensive. To address this problem, we develop a sequential design framework for HTSs. That is, we investigate the question whether the currently available data in a typical HTS suffices and, if not, how to determine the optimal stopping strategy. We focus on experiments to perform group comparisons, although our ideas remain useful for other inferential goals. For simplicity, we discuss the two-group case, but our software allows >2 groups. The proposal is based on Bayesian decision theory, so that decisions are coherent with respect to an underlying utility function and probability model. We emphasize ease of interpretation and use.

Several authors proposed fixed sample-size calculations for HTSs, i.e. the sample size is fixed at the beginning of the experiment (Dobbin and Simon, 2007; Lee and Whitmore, 2004; Müller *and others*, 2004; Pan *and others*, 2002; Zien *and others*, 2003). The main limitations are the lack of flexibility to incorporate new data and the need for a good prior guess of certain features, e.g. effect sizes or the proportion of differentially expressed (DE) genes.

In contrast, sequential sample-size designs update knowledge and make decisions as data are collected, i.e. they are robust with respect to prior choices. A sequential design stops or continues experimentation on the basis of all available data. A potential drawback is the need to carry out experimentation in batches. The associated increase in time or experimentation costs may outweigh the potential advantages. This should not be a major concern in many HTSs, as most high-throughput technologies (e.g. microarrays, sequencing, or mass spectrometry) process samples in small batches. Assessing the promise of continuing experimentation after each batch seems natural. Also, samples may be costly to obtain or there may be ethical concerns, e.g. in human studies. These situations offer great potential for sequential strategies.

 Ruppert *and others* (2007), Tibshirani (2006), and Ferreira and Zwinderman (2006) proposed two-step designs focused on microarray differential expression problems. Two-step designs adapt to the observed data to a limited extent. Gibbons *and others* (2005) and Durrieu and Briollais (2009) propose sequential designs that select a single/few genes and stop the trial when differences in expression can be estimated with high precision. The focus on a few genes limits the application to HTS. Researchers typically use HTS as a screening test to identify candidates, which are then validated with more precise techniques (e.g. real-time PCR). The usual goal is not to estimate differential expression accurately but to find promising targets. The Durrieu and Briollais (2009) model is appropriate for paired observations, e.g. two-channel arrays.

We propose an approach for unpaired data that screens a large number of candidates and attempts to maximize the number of promising targets. The framework is directly applicable to many probability models and experiments, including sequencing, microarrays, and reverse-phase protein arrays (RPPAs). With minor modifications, it can be adapted to other experimental goals. The main hurdle with decision-theoretic optimal sequential designs is the prohibitive computational cost, even in single-outcome experiments. Rossell *and others* (2006) developed an approach based on the ideas of Müller *and others* (2006), Brockwell and Kadane (2003), and Carlin *and others* (1998). They compute a summary statistic *S* each time new data are observed and they use decision boundaries that partition the sample space. The experiment is terminated when *S* first falls in the stopping region. The sequential problem is reduced to the (non-sequential) problem of finding optimal boundaries. The choice of these boundaries accounts for all future data, which distinguishes the solution from myopic approximations. Here we extend these ideas to high-dimensional data and apply them to differential expression problems.

Section 2 formalizes the problem and two convenient probability models. Section 3 describes sequential stopping and the infeasibility of an exact solution. Section 4 proposes an approximate solution. Section 5 presents examples and Section 6 some concluding remarks. The supplementary material available at *Biostatistics* online contains theoretical and practical considerations, and an example with the R code.

## 2. Data format and model

We motivate the discussion in the context of experiments that study differential gene expression, but the proposal remains applicable to other setups. Let *n* be the number of outcomes (e.g. genes) and *T* be the maximum sample size. *T* is usually determined by budget constraints, accrual rates, or an informed guess. Let *x*_*ij*_ be the measurement for gene *i*=1,…,*n* and sample *j*=1,…,*T*, and *z*_*j*_∈{0,…,*n*_*z*_} be the group of sample *j*. For simplicity, here we assume *z*_*j*_∈{0,1}, i.e. we compare two groups. Generalization to *n*_*z*_>1 is straightforward and is implemented in the gaga package.

A latent variable *δ*_*i*_=1 indicates that gene *i* is DE across groups and *δ*_*i*_=0 that it is equally expressed (EE). The indicator *δ*_*i*_ represents the unknown truth and is part of the parameter vector. Let ***θ***_*i*_ be parameters indexing a probability model for (*x*_*i*1_,…,*x*_*iT*_). Optionally, let ***ω*** be additional hyper-parameters. For example, ***ω*** could index a regression on important covariates. Let **x**_*t*_={*x*_*it*_,1≤*i*≤*n*} be the data obtained at time *t* and **x**_1:*t*_={*x*_*ij*_,1≤*i*≤*n*,1≤*j*≤*t*} be all data available up to time *t*. Further, let ***θ***=(***θ***_1_,…,***θ***_*n*_) and ***δ***=(*δ*_1_,…,*δ*_*n*_).

### 2.1 Probability model

Our proposal requires extensive predictive simulation and model fitting. Hence, the model must be computationally efficient. For instance, the examples in Section 5 required posterior inference in millions of simulated datasets. On the other hand, the model needs to be sufficiently flexible to capture the important features of the data.

Here we use the GaGa (Rossell, 2009) and log-normal normal with generalized variance (NN) models (Yuan and Kendziorski, 2006) models to illustrate the approach. Both offer a reasonable compromise between flexibility and computational cost. The GaGa model assumes *x*_*ij*_∼*Ga*(*α*_*i*_,*α*_*i*_/*λ*_*iz*_*j*__). The NN model uses 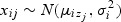. The triple ***θ***_*i*_=(*λ*_*i*0_,*λ*_*i*1_,*α*_*i*_) (GaGa) or 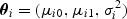 (in the NN model) incorporates gene-specific variability and gene-by-group specific means. A hierarchical prior on ***θ***_*i*_ assigns positive prior probability to means being equal across groups. The GaGa hierarchical prior is
(2.1)
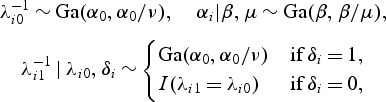

and *P*(*δ*_*i*_=1)=*π*, independently across *i*. The hyper-parameters are ***ω***=(*α*_0_,*ν*,*β*,*μ*,*π*). The NN hierarchical prior is 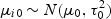, 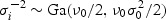, also with probability of ties *P*(*δ*_*i*_=1)=*π*, independently across *i*. For this model, ***ω***=(*μ*_0_,*τ*_0_,*ν*_0_,*σ*_0_,*π*). The GaGa sampling distribution for *x*_*ij*_ captures asymmetries that are frequently observed in HTS. The NN assumptions are similar to those of the popular limma approach (Smyth, 2004), which has been found useful in many applications. The supplementary material available at *Biostatistics* online (Section 3) shows goodness-of-fit assessments that help choose the most appropriate model for a particular dataset.

In terms of computational complexity, conditional on ***ω*** the posterior distributions are available in closed form. We treat ***ω*** as fixed, avoiding the need for Markov Chain Monte Carlo (MCMC) simulation. This substantially increases the computational speed. We estimate ***ω*** via expectation-maximization as in Rossell (2009) and Yuan and Kendziorski (2006). The latter proposed a method of moments estimate for 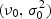, which can result in overestimating *π*. We illustrate this issue and outline a simple procedure to adjust 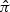 in the supplementary material available at *Biostatistics* online (Section 4).

While we use these two models in our examples, the upcoming discussion of the optimal stopping policy remains valid for any alternative probability model.

### 2.2 Pre-processing

We assume that the data are suitably pre-processed. This is critical for meaningful inference. For instance, ignoring batch effects may bias or add uncertainty to group comparisons. We note that some technologies such as RNA-seq may be less sensitive to batch effects, and that these can be partially mitigated by good design, e.g. by balancing the number of samples in each group and batch. We recommend jointly pre-processing data after every batch, as some technical biases (e.g. probe or GC-content biases) may be better assessed once more data are collected.

Batch effects and other sources of variability may be either addressed in the pre-processing or in the analysis by including appropriate terms in the model. Following Yang and Speed (2002) and Durrieu and Briollais (2009), we argue in favor of the former. As an illustration, let **y**_*ij*_ be a vector of covariates that are used in the adjustment, and assume that *E*(*x*_*ij*_|*μ*_*iz*_*j*__,**y**_*ij*_)=*μ*_*iz*_*j*__+*g*(**y**_*ij*_). Here, *g*(⋅) captures the effect of **y**_*ij*_ on the outcome, and could represent a non-linear adjustment that cannot be captured by the analysis model. One could then use the partial residuals 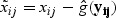 as the pre-processed data, where 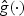 is an appropriate estimate of *g*(⋅).

We note that the domain of the data must match the assumptions of the model. For example, while most technologies deliver positive expression measurements, pre-processed data may sometimes present negative values that are not allowed in the GaGa model. A simple strategy to deal with negative values is to define 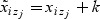, where the offset *k*>0 ensures that 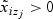. Alternatively, define 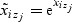, but this option may produce outliers that decrease the model goodness-of-fit. In practice, we recommend trying several transformations and producing some goodness-of-fit plots (e.g. see supplementary material available at *Biostatistics* online, Section 3).

## 3. Optimal sequential stopping

### 3.1 Decision criterion

We formalize sequential sample-size calculation within a Bayesian decision-theoretic framework. The optimal design is chosen by maximizing the expectation of an appropriate utility function. At each decision time, the expected utility is conditional on all available data (which may be no data at all) and averaged with respect to uncertainty on the model parameters and future data, assuming optimal future decisions.

It is convenient to distinguish sequential and terminal decisions. Sequential decisions correspond to stopping vs. continuation and are made after each batch of observations. Terminal decisions are the classification of genes into EE (*δ*_*i*_=0) or DE (*δ*_*i*_=1), and are taken only upon stopping. Let *s*_*t*_=*s*(**x**_1:*t*_)=1 indicate the sequential decision of stopping at time *t* and let *s*_*t*_=0 indicate continuation. Equivalently, *s*_*t*_ can be described by the stopping time 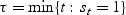. We use *s*_*t*_ and *τ* interchangeably. Let *d*_*i*_(**x**_1:*t*_)=1 (0) indicate the terminal decision to report gene *i* as DE (EE). Also, let **d**(**x**_1:*t*_)=(*d*_1_(**x**_1:*t*_),…,*d*_*n*_(**x**_1:*t*_)). Both *s*_*t*_ and **d**(**x**_1:*t*_) depend on all data available up to time *t*.

In a fully decision-theoretic approach sequential and terminal decisions are chosen to jointly maximize the expected utility. Instead, we assume a fixed rule for **d**(**x**_1:*t*_) and focus on the optimal choice of *s*_*t*_ only. We take terminal decisions using the Bayes rule of Müller *and others* (2004) to control the posterior expected false discovery rate (FDR) below some specified level. The posterior expected FDR is 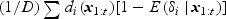, where 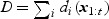 is the number of reported positives. We use the 0.05 level throughout.

Sequential stopping decisions *s*_*t*_ are based on a utility function with sampling cost *c* and a unit reward for each correctly identified DE outcome
(3.1)
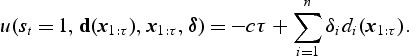

The second term in (3.1) is the number of true positives (TPs). The cost *c* is the minimum number of TPs that make it worthwhile to obtain one more sample. This interpretation allows for easy elicitation of *c*, without any reference to the formal mathematical framework. The utility function (3.1) focuses on statistical rather than biological significance, as the size of the effect is not considered. A simple alternative is obtained by substituting |*μ*_*i*1_−*μ*_*i*2_|*δ*_*i*_*d*_*i*_(***x***_1:*τ*_) in the summation in (3.1); see Müller *and others* (2004) or Rice *and others* (2008) for other interesting alternatives. The upcoming discussion is independent of the specified utility.

### 3.2 Optimal rule

The optimal stopping decision *s*_*t*_ maximizes *u*(⋅), in expectation over all unknowns, including parameters (***θ***,***δ***), and future data **x**_*τ*+1:*T*_. An exact solution requires dynamic programming, also known as backward induction (DeGroot, 2004). At time *t*, the optimal decision is to stop if the posterior expected utility for *s*_*t*_=1, denoted by 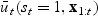, is greater than 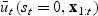. Evaluating 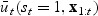 is usually straightforward. For (3.1), we find
(3.2)
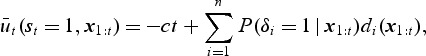

where *P*(*δ*_*i*_=1|**x**_1:*t*_) is the posterior probability that outcome *i* is DE. The expectation is with respect to ***δ*** only, as we fix the terminal decision **d**(**x**_1:*t*_). The posterior probability *P*(*δ*_*i*_=1|***x***_1:*t*_) can be computed in closed form for some models (including GaGa and NN) or can easily be estimated from the MCMC output. Evaluating 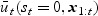 is more challenging. An exact solution requires assessing the expected utility for all possible future data trajectories ***x***_*t*+1_,…,***x***_*T*_, substituting the optimal decisions *s*_*t*+1_,…,*s*_*T*_. The computational cost is prohibitive.

## 4. Approximation by optimal decision boundaries

 Berry *and others* (2001), Brockwell and Kadane (2003), DeGroot (2004), and Müller *and others* (2006) discuss alternatives to an exact optimal sequential solution. Following Rossell *and others* (2006), we define sequential stopping boundaries. We restrict the maximization to rules that depend on the data *x*_1:*t*_ only through a summary statistic *S*_*t*_ and linear boundaries that partition the sample space. We propose using *S*_*t*_=Δ_*t*_U, where

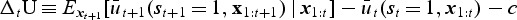

is the one-step ahead increase in the expected utility and *E*_**x**_*t*+1__(⋅|**x**_1:*t*_) conditions on **x**_1:*t*_ and marginalizes with respect to future data **x**_*t*+1_. For (3.1), we find Δ_*t*_U=Δ_*t*_(TP)−*c*, i.e. Δ_*t*_U is the expected increase in the TPs, and decision boundaries can equivalently be written in terms of Δ_*t*_(TP).

**Fig. 1.**
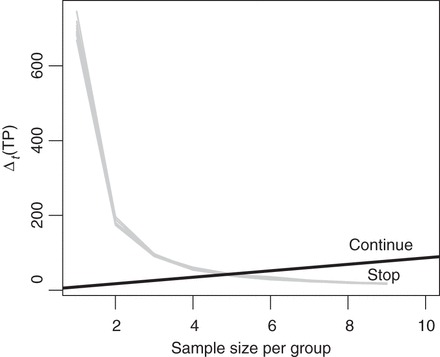
A GaGa model based optimal sequential boundary for *c*=50 (thick black line) and forward simulation trajectories (light gray lines), for example, in Section 5.1.

Consider the example in Figure 1. The thick black line is a decision boundary. Every time we observe new data, we compute Δ_*t*_(TP). If Δ_*t*_(TP) lies above the boundary, we continue experimentation, otherwise we stop. That is, we experiment as long as enough new TPs are expected.

Let ***b***=(*b*_0_,*b*_1_) be the intercept and slope defining the linear boundaries, and U(***b***,***x***_1:*t*_) be the associated expected utility given data up to time *t*. In other words, U(***b***,***x***_1:*t*_) is the expected utility conditional on ***x***_1:*t*_ when the stopping decision is based on a decision boundary indexed by ***b***. Algorithm 1 details a forward simulation algorithm (Carlin *and others*, 1998) to evaluate the required expectations, and a grid search to carry out the maximization of U(***b***,***x***_1:*t*_) with respect to ***b***. The algorithm assumes that *t* samples are already available. For no data use *t*=0 and ***x***_1:*t*_=∅ (see Section 5.3).

**Algorithm 1**

Optimal sequential boundary determination.

1. *Forward simulation*. Simulate 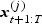 from the posterior predictive *P*(***x***_*t*+1:*T*_|***x***_1:*t*_), *j*=1,…,*B*. For each 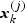, compute Δ_*t*_(TP)^(*j*)^, *k*=*t*+1,…,*T*.
2. *Grid search.* For each ***b***, find the stopping times *τ*^(*j*)^ for all saved trajectories Δ_*t*_(TP)^(*j*)^.
3. *Optimum*. Select 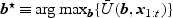, where 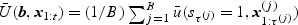.

Figure 1 shows simulated Δ_*t*_(TP) as gray lines. For each boundary ***b***, we determine the stopping time for each trajectory and average the expected terminal utilities. At *t*=*T*−1, we do not determine stopping using ***b*** but the optimal rule Δ_*T*−1_U>0.

In principle ***b***^★^ can be recomputed every time new data are observed. Recomputation can help to decide between multiple optima and update *P*(***x***_*t*+1:*T*_|***x***_1:*t*_). In our examples, we determine ***b***^★^ only once, either based on a pilot dataset or prior knowledge, but we indicate the usefulness of recomputation when appropriate.

In addition to the intuitive appeal, some theoretical considerations motivate our approach. First, fixed-sample designs are special cases, e.g. 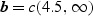 results in a fixed sample size of 5. The myopic rule of continuing as long as Δ_*t*_U>0 (Berry and Fristedt, 1985, Chapter 7) is the special case ***b***=(*c*,0). We generalize the idea with an arbitrary boundary on Δ_*t*_U. An important assurance is that Δ_*t*_TP converges to 0 as 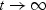, which guarantees eventual stopping; see supplementary material available at *Biostatistics* online (Section 1) for a formal statement and proof.

## 5. Examples

We compare our approach and the fixed sample designs of Müller *and others* (2004) in several important experimental conditions. The supplementary material available at *Biostatistics* online discuss pre-processing and goodness-of-fit (Section 3) and an additional RNA-seq example with the R code (Section 4).

### 05.1 Simulated microarray study

We plan collecting data in batches of 2 arrays per group, with a maximum of 20 per group (i.e. *T*=10 batches). Recall that *c* is the minimum number of new DE genes that compensate the cost of one more batch. We consider *c*=25, 50, and 100. To keep the simulation realistic, we estimated the hyper-parameters based on data from a study of leukemia microarray data (Armstrong *and others*, 2002). We focus on 24 acute lymphoblastic leukemia (ALL) and 18 mixed-lineage leukemia (MLL) trans-location samples. The estimated proportion of DE genes is 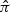
=0.063 under the GaGa model and 0.05 for the NN model. We find optimal strategies based on 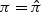, but we assess performance under model mis-specification by also simulating data under 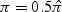 and 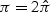, while leaving 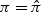 unchanged in the analysis model. We obtained 250 simulations under each scenario.

Figure 1 shows the optimal boundary for *c*=50 and simulated Δ_*t*_(TP) (gray lines) under the GaGa model. Table 1 reports expected utilities and stopping times. The optimal fixed sample sizes for *c*=50 under the GaGa and NN models are 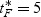 and 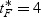, respectively. When 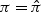, the expected sequential sample sizes are 5.0 and 4.1 (respectively) and there is no gain in the posterior expected utility. Sequential designs offer little advantages when the data match the prior expectations. However, when prior expectations are unrealistic, sequential designs adapt to the observed data. When *π* was overstated by the prior (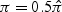), sequential designs stopped earlier than the fixed sample-size designs. Conversely, when 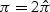, they stopped later so that more DE genes could be found. For instance, for *c*=50 the GaGa sequential design requires 4.1 data batches when 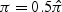 and 6.9 when 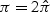. The fixed design always requires 5.

**Table 1.** Simulated data. 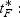 fixed sample size; 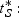 average sequential sample size; 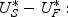 expected utility for sequential design minus the expected utility for a fixed sample

		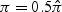		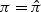		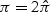	
*c*	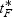	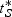	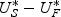	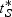	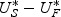	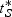	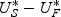
GaGa							
25	7	6.0	7.7	7.1	0.4	10.0	34.2
50	5	4.1	16.6	5.0	0.0	6.9	28.7
100	3	3.0	0.0	3.0	0.0	4.0	58.6
NN							
25	7	5.6	11.1	7.2	0	10.0	32.4
50	4	3.2	10.8	4.1	0.1	5.7	51.2
100	3	2.0	41.9	3.0	0.1	3.0	0

### 5.2 High-throughput sequencing example

We use a pilot RNA-seq dataset with two muscle and one brain human samples to design two hypothetical studies. Study 1 compares gene expression for muscle vs. brain. Many DE genes are expected. Hypothetical Study 2 compares the two muscle samples. No genes should be DE. In both cases, we use one sample per group as pilot data. We consider up to *T*=5 more samples, in batches of one sample. The GaGa model provided a reasonable fit to these data (supplementary material available at *Biostatistics* online, Section 3).

**Fig. 2.**
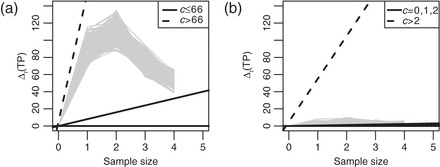
Simulated one-step expected increase in true discoveries Δ_*t*_(TP) (gray lines) and optimal boundaries for several sampling costs *c* (black lines). Left: brain vs. muscle (two multiple optimal boundaries shown for *c*≤66), right: muscle vs. muscle.

We determined the optimal boundary for sampling costs *c*=0,1,…,100. Figure 2(a) shows that Δ_*t*_(TP) is maximal for *t*=2 additional data batches. As suggested by Theorem 1 (supplementary material available at *Biostatistics* online, Section 1), the incremental reward decreases as *t* grows further. The dashed boundary shows that, for *c*>66, the optimal decision is to stop experimentation. For *c*≤66, there are multiple optimal b*. The solid black lines show two optima. In both cases, the decision at *t*=0 is to continue. Since the simulated trajectories do not cross either boundary, we expect experimentation to continue up to *T*=5. The future realized Δ_*t*_(TP) might cross the boundary, in which case the design would adapt and stop experimentation before *T*=5. Given that the pilot data contains one sample per group, we would re-determine ***b***^★^ upon observing new data.

The hypothetical muscle vs. muscle comparison simulation is shown in Figure 2(b). In this case, Δ_*t*_(TP) is negligible and the optimal design is to stop at *t*=0 (i.e. not to collect any further data) for any *c*>2. The result seems sensible as no DE genes are expected.

### 5.3 Microarray example

We consider the leukemia study of Campo Dell'Orto *and others* (2007) recording mRNA expression for 21 ALL and 15 MLL patients and 54 675 genes. We consider designing the study before any data were available. In such circumstances, one could estimate the hyper-parameters ***ω*** from a similar study. We used the Armstrong *and others* (2002) study (Section 5.1) as it was also carried on ALL/MLL patients and used the same microarray platform. Once fixed and sequential designs were determined, we used the historical data to compare performance. We use batches of two arrays per group, maximum *T*=7 batches, and *c*=50.

**Fig. 3.**
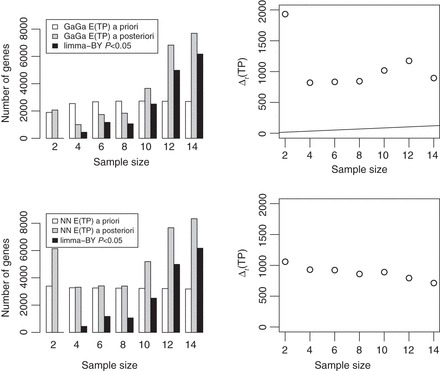
Sequential Analysis of Campo Dell’Orto’s data based on GaGa (top panels) and NN models (bottom panels). Left panels: expected number of TP (a priori and a posteriori) vs. sample size. Black bars indicate the number of genes with Benjanimi–Yekutieli-adjusted limma *P*-values <0.05. Right panels: Δ_*t*_(TP) vs. sample size and optimal sequential boundary. Δ_*t*_(TP) being above the boundary for all *t* indicates the experimentation continuing up to the maximum sample size.

The white bars in Figure 3 (left panels) show the expected utility under the GaGa and the NN priors for all fixed sample sizes. The optimal fixed sample sizes are 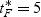 batches (GaGa) and 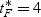 (NN). The right panels show the optimal boundaries and Δ_*t*_(TP) computed from the observed data up to time *t*=1,…,7. For both models, the sequential design continues up to the time horizon. Figure 3 compares the designs by computing the posterior expected TPs (gray bars) and the number of genes with limma *P*-values <0.05 after the Benjamini and Yekutieli (2001) adjustment (black bars). At the time horizon both quantities increase over 2-fold compared with the recommended fixed sample size. The differences between prior and posterior expected TPs show how sequential designs adapt to the observed data to correct prior mis-specifications.

### 5.4 Reverse-phase protein arrays

We design a follow-up study for the RPPA dataset dataIII that is included in the R package RPPanalyzer (Mannsperger *and others*, 2010). The data contain expression for 75 proteins and 35 stage 2 and 25 stage 3 samples. Both models, the NN and GaGa models provide a reasonable fit (supplementary material available at *Biostatistics* online, Section 3). The fit under the NN model is slightly better. We find 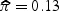 under the NN model, and 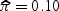 under the GaGa model; that is, the estimated number of DE proteins is 9.75 and 7.5, respectively. Although we expect several DE proteins, at a posterior expected FDR <0.05 the NN model calls one DE protein, and the GaGa model makes no DE calls. For comparison, only one protein has limma BY-adjusted *P*-values below 0.05.

**Fig. 4.**
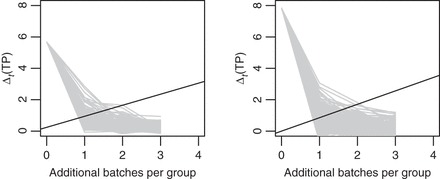
Simulated Δ_*t*_(TP) and optimal boundaries for *c*=1 in RPPA data using GaGa (left) and NN (right) models.

We consider adding batches of 50 samples per group, up to a maximum of *T*=4. We set the sampling cost to *c*=1, reflecting that RPPA samples are relatively cheap. The study focuses on 75 carefully chosen proteins. Figure 4 shows simulated Δ_*t*_(TP) trajectories and the optimal boundaries. Inference under the GaGa model estimates fewer TPs. Otherwise, inference is fairly similar across models and the optimal boundaries are remarkably close. The average sample size is 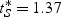 under the NN model, and 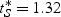 under the GaGa model. The expected number of TPs at 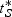 is 9.46 (NN) and 6.11 (GaGa); that is, according to both models, most DE proteins should be detected by adding 1–2 batches, i.e. 50–100 samples per group. These results help assess the potential benefits of extending the experiment.

## 6. Discussion

We proposed a sequential strategy for massive multiple hypotheses testing. An important advantage lies in the generality of the proposed design. We discussed three RNA-seq, one microarray, and one RPPA experiments. Sequential designs are robust with respect inaccurate prior guesses and provide substantial advantages over fixed sample designs.

The proposal is formulated in a decision-theoretic framework and emphasizes interpretability. We monitor the one-step ahead expected increment in utility and stop the experiment when it falls below a boundary. The approach includes fixed sample size and myopic designs as special cases. We use terminal decisions that control the posterior expected FDR. While inconsistent with a strict decision-theoretic setup, where all decisions are taken to maximize the expectation of a single utility, we feel that our choice offers a pragmatic compromise.

The method allows stopping when only one or two samples are available, which requires making strong parametric assumptions. For instance, in Figure 3, the posterior expected TPs and Δ_*t*_(TP) based on two samples per group differ widely between the GaGa and NN models. Nevertheless, both models correctly indicate to continue and show good agreement for subsequent samples. Whenever possible, we recommend using a minimum burn-in (e.g. ≥3 samples) before starting sequential stopping. When not feasible, we recommend assessing the goodness-of-fit carefully and updating the forward simulation when more data are available.

We focused on group comparison experiments, but the framework can serve as the basis for other HTSs. Interesting extensions include classification, clustering, or network discovery studies. These would require adjusting the utility function and possibly the probability model, e.g. to capture strong dependencies between outcomes.

Sequential designs are most appealing in moderate to large studies, where technical limitations require gathering data in batches. They should also prove valuable when samples are costly to obtain or there are ethical considerations, e.g. in human studies. Overall, they help save valuable resources and guarantee that sufficient data are collected to answer the scientific questions.

## Software

An implementation of the proposed approach was added to the Bioconductor package gaga.

## Supplementary material

Supplementary material is available at http://biostatistics.oxfordjournals.org.

## Funding

P.M. was partially supported by NIH grant R01 CA075981.
